# Dendritic mGluR2 and perisomatic Kv3 signaling regulate dendritic computation of mouse starburst amacrine cells

**DOI:** 10.1038/s41467-024-46234-7

**Published:** 2024-02-28

**Authors:** Héctor Acarón Ledesma, Jennifer Ding, Swen Oosterboer, Xiaolin Huang, Qiang Chen, Sui Wang, Michael Z. Lin, Wei Wei

**Affiliations:** 1 Graduate Program in Biophysical Sciences, The University of Chicago, Chicago, IL 60637 USA; 2 The Committee on Neurobiology Graduate Program, The University of Chicago, Chicago, IL 60637 USA; 3 The Committee on Computational Neuroscience Graduate Program, The University of Chicago, Chicago, IL 60637 USA; 4 Department of Ophthalmology, Stanford University, Stanford, CA 94305 USA; 5 Department of Neurobiology, Department of Bioengineering, Stanford University, Stanford, CA 94305 USA; 6 Department of Neurobiology and the Neuroscience Institute, The University of Chicago, Chicago, IL 60637 USA; 7 Present Address: F.M. Kirby Neurobiology Center, Boston Children’s Hospital, Harvard Medical School, Boston, MA 02115 USA; 8 Present Address: Department of Neurobiology, Harvard Medical School, Boston, MA 02115 USA; 9 Present Address: Division of Neurobiology, Department of Molecular and Cell Biology, Helen Wills Neuroscience Institute, University of California, Berkeley, CA 94720 USA; 10 Present Address: Department of Physiology & Biophysics, University of Washington, Seattle, WA 98195 USA

**Keywords:** Ion channels in the nervous system, Retina, Dendritic excitability, Membrane potential, Sensory processing

## Abstract

Dendritic mechanisms driving input-output transformation in starburst amacrine cells (SACs) are not fully understood. Here, we combine two-photon subcellular voltage and calcium imaging and electrophysiological recording to determine the computational architecture of mouse SAC dendrites. We found that the perisomatic region integrates motion signals over the entire dendritic field, providing a low-pass-filtered global depolarization to dendrites. Dendrites integrate local synaptic inputs with this global signal in a direction-selective manner. Coincidental local synaptic inputs and the global motion signal in the outward motion direction generate local suprathreshold calcium transients. Moreover, metabotropic glutamate receptor 2 (mGluR2) signaling in SACs modulates the initiation of calcium transients in dendrites but not at the soma. In contrast, voltage-gated potassium channel 3 (Kv3) dampens fast voltage transients at the soma. Together, complementary mGluR2 and Kv3 signaling in different subcellular regions leads to dendritic compartmentalization and direction selectivity, highlighting the importance of these mechanisms in dendritic computation.

## Abstract (web summary)

How starburst amacrine cell (SAC) dendrites transform concentrically distributed synaptic inputs into branch-specific directional outputs is not fully understood. Here the authors report that dendritic mGluR2 signaling and somatic Kv3-mediated shunting coordinately implement SAC dendritic direction selectivity.

## Introduction

Dendrites profoundly shape the input-output relationship of a neuron. In particular, compartment-specific processing of synaptic signals has been shown to confer additional computational power of dendrites^1–3^. Because of the intricate dendritic morphology and non-uniform distribution of synaptic inputs and ion channels on dendrites, synaptic signals undergo specific transformations in different dendritic subregions. A compelling example of such dendritic computation is the implementation of direction selectivity by the dendrites of starburst amacrine cells (SACs).

**Fig. 1:**
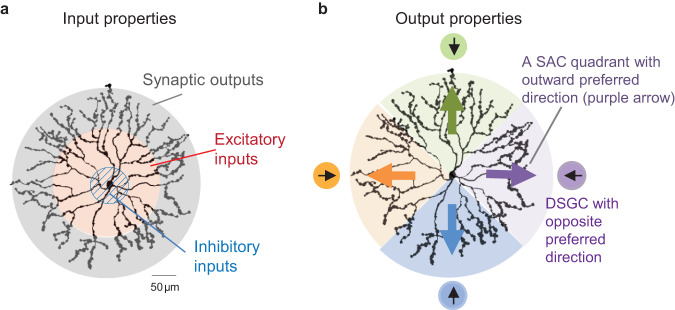
Different divisions of SAC dendritic compartments based on synaptic input and output properties. **a** Synaptic inputs and outputs are differentially enriched along the radial dendritic axis. **b** The output of SACs is divided into four dendritic quadrants that exhibit different preferred motion directions and inhibit distinct postsynaptic direction-selective ganglion cells (DSGCs).

The SAC in the mammalian retina is an axon-less interneuron with a radially symmetric dendritic arbor that narrowly stratifies in either the On or Off sublamina of the inner plexiform layer (IPL)^4–6^. In the mouse retina, synaptic inputs and outputs of a SAC are segregated into different concentric dendritic zones around the soma^7,8^ (Fig. 1a): synaptic inputs are concentrated in the proximal two thirds of the radiating dendritic branches, while synaptic release of GABA and acetylcholine (ACh) occurs in the distal third of the dendrites from specialized structures called varicosities^9^. Synaptic outputs from individual dendrites are direction-selective to the outward motion direction for a given branch (from soma to tip, or the centrifugal direction) (Fig. 1b). A moving stimulus over the SAC receptive field strongly activates distal varicosities from the SAC dendritic branch oriented in the direction of motion, but varicosities in the opposite branch of the same cell show minimal activation^7,8,10–13^.

Outward direction selectivity of SAC synaptic release is critical for the direction selectivity of their postsynaptic partners, On- and On-Off types of direction-selective ganglion cells (DSGCs)^14^. DSGCs can be divided into four subtypes based on their preferred motion directions along the vertical and horizontal axes^15,16^. Each SAC dendritic quadrant selectively forms GABAergic synapses with one of the four DSGC subtypes so that presynaptic SAC dendrites are oriented in the null direction of the postsynaptic DSGC^17–21^ (Fig. 1b). Strong GABA release from outward-activated SAC dendrites generates strong null-direction inhibition of DSGCs, a key mechanism underlying the direction selectivity of DSGC spiking response.

How SAC dendrites transform concentrically distributed synaptic inputs into branch-specific directional outputs is an exemplary question of dendritic computation. A reductionist approach to study this question is to focus on the direction-selective dendritic integration within a sector from soma to tip, under the assumption of independent motion processing by individual radial branches. This useful approach has led to the discovery of multiple local mechanisms of the outward direction selectivity within a dendritic branch, such as the segregation of proximal inputs and distal outputs^7,8^, differential kinetics^22–25^ ^but see^^7,8,26,27^ and center surround receptive field properties of excitatory inputs^27–29^ in proximal and medial parts of the dendrite, and lateral inhibition from neighboring SACs^8,11,13,30,31^.

Despite the independent output properties of SAC dendrites such as their direction preference and postsynaptic targeting, motion signals in dendrites may not solely originate from local synaptic inputs within that sector, because all dendritic sectors are connected by the soma and are thus not completely electrotonically isolated. In fact, proximal dendrites near the soma contain abundant excitatory synaptic inputs^8^, and therefore represents a converging point for motion signals arising from multiple sectors to meet, summate and spread along the dendritic tree. To maintain the outward direction selectivity of distal dendrites, the SAC must be equipped with mechanisms that tightly regulate the interaction between local synaptic signals and those originating from distant dendritic sectors. Computational modeling suggests that passive membrane properties provide partial electrotonic isolation between sectors to maintain sector-specific directional tuning^32,33^. Voltage-dependent mechanisms are also implicated in SAC dendritic computation^10,34,35^. SAC proximal dendrites are enriched in voltage-gated potassium channels Kv3, which have been proposed to function as a voltage-dependent shunt to limit depolarization near the soma^36^. Furthermore, mGluR2 signaling in SACs, which inhibits voltage-gated N and P/Q type voltage-gated calcium channels (VGCCs), is required for maintaining outward direction selectivity of individual sectors^12^. However, it is unclear how these dendritic machineries collectively transform synaptic inputs across the dendritic field into branch-specific outputs during visual motion processing.

Here, using a combination of two-photon subcellular calcium and voltage imaging, and patch clamp recording, we investigated the temporal relationship between the global motion signal arising from distal synapses and local synaptic inputs onto a dendritic branch in the inward versus outward motion direction. Furthermore, we found that the coordinated action of mGluR2 and Kv3 signaling confers distinct modes of dendritic integration at the perisomatic and dendritic regions: (1) Kv3 is required for maintaining a steady depolarization at the soma during the summation of motion-evoked synaptic inputs across dendrites, and (2) mGluR2 is required for modulating dendritic VGCCs to generate direction-selective, suprathreshold calcium transients in the outer half dendritic zone. These results highlight the importance of metabotropic signaling and voltage-dependent membrane conductances in the functional compartmentalization of neuronal dendrites.

## Results

### A moving bar across the SAC receptive field causes accumulating depolarization at the soma representing a low pass filtered global motion signal

The high density of synaptic inputs onto SAC proximal dendrites^7,8^ alludes to an important role of the perisomatic region as a central hub where synaptic inputs from multiple sectors converge and interact. Therefore, we first examined the spatiotemporal relationship between synaptic inputs and somatic membrane potential (Vm) in response to a moving stimulus. We stimulated whole-mount retinas with a moving bright bar in the photopic range focused on the outer segments of photoreceptors (W × L: 275 μm × 440 μm). Excitatory postsynaptic currents (EPSCs) or somatic Vm of On SACs were measured by whole-cell patch clamp recording.

**Fig. 2:**
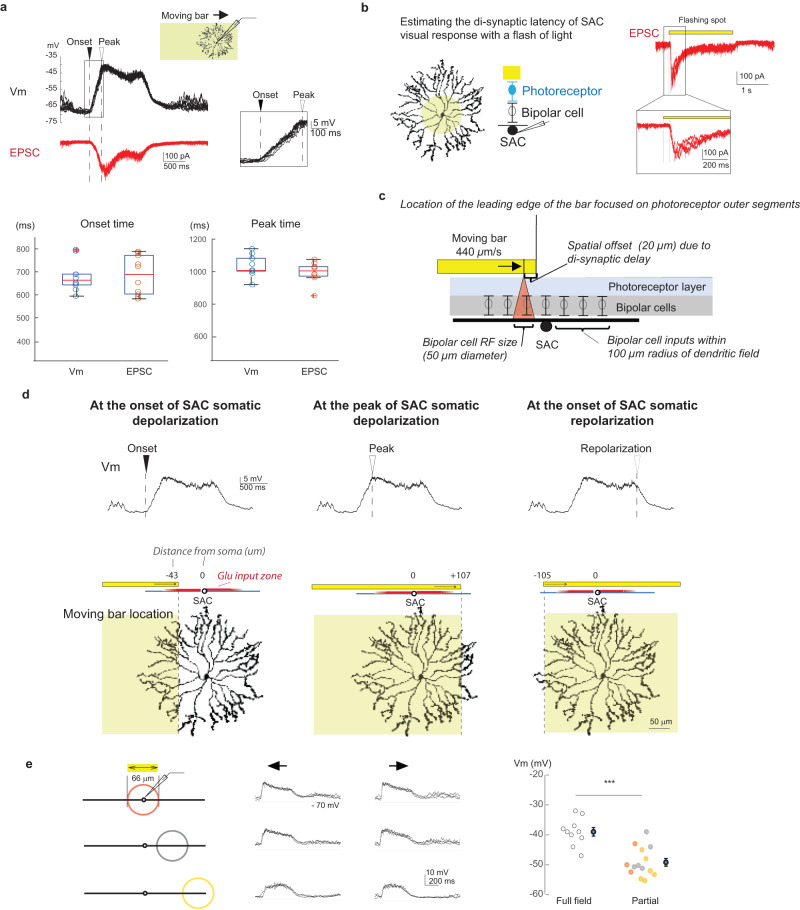
A global motion signal at the soma reflects accumulating depolarization during moving bar stimulation. **a** Upper: Current clamp (black) and voltage clamp (red) recording traces of SACs during the moving bar stimulus (yellow box on top). Arrowheads indicate the onset and peak of Vm and EPSC light responses. Inset shows Vm trace during the depolarizing phase in expanded time scale. Traces from 10 trials are overlayed. Vertical dashed lines are for comparison between Vm and ESPC traces in the time axis. Lower: Onset and peak times of Vm and EPSC traces. Two-sided student *t* test onset time *p* = 0.78; peak time *p* = 0.23. Vm, 6 cells; EPSC, 10 cells. For each box, the central mark indicates the median, and the bottom and top edges of the box indicate the 25th and 75th percentiles, respectively. The whiskers extend to the most extreme data points not considered outliers, and the outliers are plotted individually using the ‘+‘ symbol. **b** SAC ESPC traces (overlay of 4 trials) in response to a flashing light spot (yellow circle on top schamtic, yellow bars above traces). Inset shows the latency of EPSC response to the flash in expanded time scale. **c** Relationship between the retinal location of the moving bar edge and the SAC dendritic location receiving edge-evoked bipolar cell inputs. (See text for more details). **d** Estimated retinal location of the moving bar (yellow shaded region) at the onset of somatic Vm depolarization (left), the peak of depolarization (middle) and at the onset of Vm repolarization (right). Locations are based on mean values of the Vm and EPSC datasets. **e** Left: schematics showing the spatial extent (circles) of the moving bar stimuli. Middle: example Vm traces in response to local moving bar stimuli (overlay of 3 trials). Right: Vm of the peak response under different stimulus conditions. ***p = 2.8 × 10^5^, two-sided unpaired student *t* test, full-field 10 cells; partial stimulation 14 cells. Data are presented as mean values ± SEM. Source data are provided as a Source Data file.

As the leading edge of the bar enters the SAC receptive field surround, somatic Vm first exhibits reduced spontaneous activity and hyperpolarization due to lateral inhibitory inputs from neighboring amacrine cells^8,30,37,38^, followed by a depolarizing phase as the bar activates bipolar cell-mediated excitatory inputs in the receptive field center (Fig. 2a). The EPSC waveform matches the somatic Vm waveform during the depolarizing phase. Furthermore, the onset time of the Vm depolarization coincides with the onset of EPSCs (Fig. 2a), indicating a predominant role of bipolar cell-mediated glutamatergic inputs in initiating and shaping the depolarizing phase.

Next, we estimated the dendritic subregion of the SAC that receives the moving bar-evoked glutamatergic synaptic inputs from bipolar cells at a given time. We first measured the synaptic transmission latency of SAC excitatory postsynaptic currents (EPSCs) in response to visual stimulation of photoreceptors. To precisely measure the synaptic release latency, we presented a 100 μm diameter flashing bright spot to the center of the On SAC dendritic field to synchronize photoreceptor activation. Under our experimental condition, light-evoked EPSCs of SACs are initiated 46 + /− 4 ms (mean, SEM, 9 cells) after the onset of the light flash (Fig. 2b), due to the di-synaptic delay between the photoreceptor activation and SAC postsynaptic response. For a bar moving at 440 μm/s across the retina, this synaptic delay corresponds to a 20 + /− 2 μm vertical spatial offset between the bar edge location on the photoreceptor surface and the resultant bipolar cell synaptic inputs on SAC dendrites. Next, we estimated the spatial divergence from the retinal surface location of the visual stimulus to bipolar cell-mediated glutamatergic inputs onto the SAC dendritic arbor, leveraging the knowledge gained from previous functional, anatomical and connectomic studies on mouse bipolar cell receptive fields. The majority of bipolar cell inputs are distributed in the proximal 100 μm of SAC dendritic branches^8^. The major bipolar cell types synapsing onto On SACs, Types 5 and 7, have dendritic and axonal arbors of ~ 30–50 μm diameter^8,39^, and RF field sizes between 40 and 50 μm^40,41^. Therefore, we estimate that the edge of the moving bar stimulus evokes glutamatergic inputs from a row of bipolar cells onto a dendritic subregion of ~50 μm in width behind the bar edge over the inner 100 μm-radius circle of SAC dendritic field (Fig. 2c).

Estimating the spatiotemporal relationship between the location of the moving stimulus and the SAC dendritic location receiving stimulus-evoked bipolar cell inputs allows us to link excitatory input pattern to the Vm response of the SAC. As the incoming bar activates an increasing number of bipolar cell inputs onto the SAC dendritic field, SAC somatic Vm response exhibits a monotonic increase in depolarization (Fig. 2a, inset). The depolarizing phase begins when the leading edge of the bar activates glutamatergic inputs onto the distal zone of the inward-activated SAC dendritic sector (Fig. 2d, left), which is consistent with the anatomical location of distal bipolar cell inputs onto SAC dendrites. Somatic depolarization peaks at the time when the bar covers the entire area of bipolar cell inputs onto the SAC (Fig. 2d, middle), and starts to repolarize as the trailing edge of the bar leaves the dendritic field (Fig. 2d, right). We never observed somatic spikes in SACs even with bars at the highest brightness and contrast levels in our visual stimulus setup. These results indicate that the SAC perisomatic region accrues the moving bar-evoked synaptic excitation over the dendritic field in the form of a monotonic increase of depolarization that is devoid of fast Vm transients. In this study, we termed this somatic Vm response “global motion signal” because it represents the accumulating excitatory inputs from the entire SAC dendrites over the motion trajectory in the form of a low pass filtered depolarization.

To further demonstrate the accumulation of dendritic depolarization at the soma, we restricted the moving bar stimulus to a 66 μm diameter aperture positioned at different dendritic locations of the SAC (Fig. 2e). We observed significant depolarization at the soma even when the stimulus is centered 110 μm from the soma. This result further shows that local depolarization by excitatory inputs across the dendritic arbor readily spreads to the soma.

### The global motion signal is present throughout SAC dendrites but coincides with local synaptic inputs differentially for inward versus outward motion directions

**Fig. 3:**
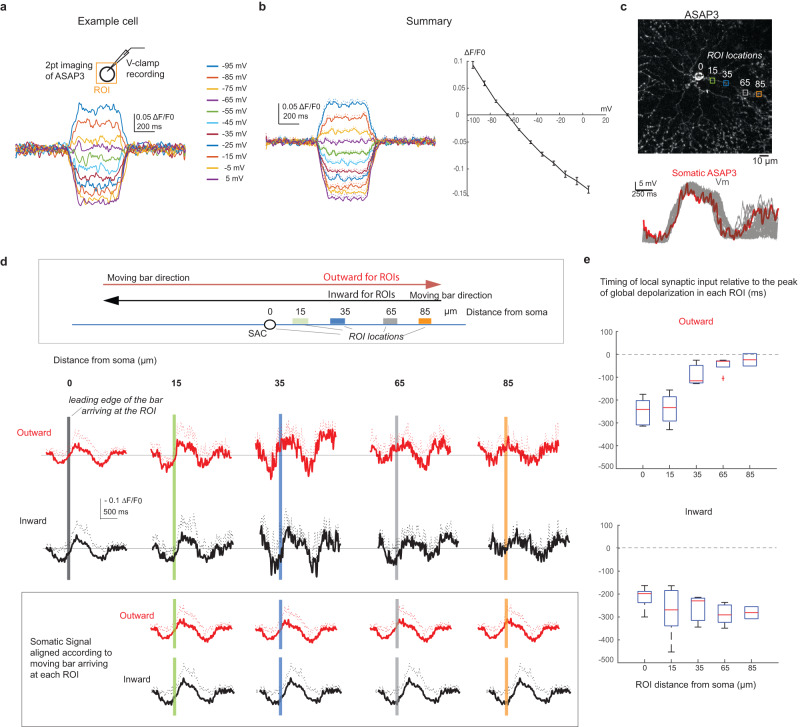
The relative timing between global and local motion signals in SAC dendrites is different for inward and outward motion directions. **a** ASAP3-Vm relationship from an ASAP3-expressiong SAC by simultaneous imaging and voltage clamp recording at the soma. **b** Summary of ASAP3 -Vm relationship measured at the soma. Left: ASAP3 traces (Mean: solid lines; SD: dashed lines, 9 cells). Right: ASAP3-Vm plot. ASAP3 signals are averaged during each voltage step. Error bars represents SEM. **c** Upper: Dendritic imaging of ASAP3 during the moving bar stimulus. ROIs (colored squares) were placed along the dendrite. Numbers indicate distances (µm) from the soma. Lower: Overlay of the average somatic ASAP waveform (red) and 20 individual trials of somatic Vm traces of a SAC (gray). ASAP3 signal is converted to voltage based on the ASAP3-Vm curve. Scale bar: 10 µm. **d** Motion-evoked ASAP3 signals are grouped into 20 µm bins centered at different radial distances. Upper: schematics of outward (red arrow) and inward (black arrow) moving bar relative to the binned imaging areas (colored rectangles). Middle: Mean (solid lines) and standard deviation (SD) (dashed lines) of ASAP3 signals in outward (red) and inward (black) motion directions. Gray solid horizontal lines represent the baseline ASAP3 signal measured between moving bar trials. Vertical colored lines indicate the time when the leading edge of the bar arrives at the imaged ROI in the upper schematic. From soma to distal bins: *n* = 5, 7, 3, 5, 2 cells. Lower: Somatic signals are aligned according to the time when the bar arriving at each dendritic ROI (colored vertical lines). **e** Summary of the relative timing between the arrival time of local synaptic inputs and the peak of ASAP3 signal for each ROI location. Negative values indicate the local synaptic inputs before the peak time of the ASAP3 signal. From soma to distal bins: *n* = 5, 7, 3, 5, 2 cells. Box plots are defined as in Fig. 2a. Source data are provided as a Source Data file.

While current clamp recordings accurately measure the global motion signal at the SAC soma, it is unknown how the global signal spreads and transforms along the dendrites. Therefore, we measured dendritic Vm responses to the moving bar stimulus using two-photon voltage imaging. Individual SACs were sparsely labeled with the genetically encoded voltage sensor ASAP3, whose fluorescence decreases upon membrane depolarization (Fig. 3). First, we characterized the ASAP3 fluorescence - Vm relationship in SACs by imaging somatic ASAP3 fluorescence while holding somatic Vm at different potentials in voltage-clamp recordings. We observed a highly consistent, near-linear ΔF/F0 – Vm relationship across multiple SACs in the −95 to −5 mV range (Fig. 3a, b), in agreement with the published characterization of ASAP3 proteins^42–44^. Next, we compared somatic Vm recordings and somatic ASAP3 fluorescence waveforms during the moving bar stimulation, and found that ASAP3 signals accurately report the shape and amplitude of subthreshold depolarization measured in current clamp recordings (Fig. 3c).

To examine the soma-to-dendrite transformation of SAC Vm, we sequentially imaged ASAP3 signals from soma toward distal dendrites of individual SACs during the moving bar stimulus (Fig. 3c, top). We averaged over multiple imaging trials for each region of interest (ROI), and combined higher frame rate with temporal smoothing (see Methods). This procedure allowed us to obtain sufficient signals to monitor slow Vm changes at the soma and into the dendrites. However, fast electrical events such as dendritic spikes were undetectable. Therefore, the ASAP3 signal in these experiments represents the low-pass filtered subthreshold component of the dendritic and somatic Vm signal, which is the “global motion signal” defined in our study.

Dendritic ASAP3 responses to the moving bar at multiple radial distances showed a depolarizing phase that is similar to that observed at the soma (Fig. 3d, signal polarity was flipped for easier comparison with the Vm waveform), indicating that bipolar cell inputs across the SAC dendritic arbor generate a global signal that spreads throughout the soma and dendrites and contributes to the local dendritic excitation. At all dendritic locations, local bipolar cell inputs occur during the depolarizing phase of the ASAP signal (indicated by the vertical colored bars in Fig. 3d). This is consistent with coincident onset of EPSCs and somatic Vm depolarization observed in current clamp recordings (Fig. 2a).

Since ASAP3 reports bidirectional Vm changes, we were able to observe that dendritic segments ~35 μm from the soma exhibited more pronounced hyperpolarization preceding the depolarization compared to those at other soma and dendritic locations (Fig. 3d). Interestingly, recent connectomic analysis shows that this dendritic subregion contains the peak density of inhibitory inputs^8^. Stronger hyperpolarization of this subregion suggests that the inhibitory control of SACs is spatially non-uniform and most effective at proximal dendrites instead of exerting a uniform influence across the dendritic field. However, since dendritic suprathreshold events are triggered during the depolarizing phase driven by bipolar cell inputs, we focused on the excitatory inputs for the rest of this study.

Although the global motion signal is present in both the somatic and dendritic membrane of the SAC during the moving bar stimulation, the relative timing between subthreshold ASAP3 waveforms and the onset of local excitatory inputs (estimated based on the moving bar location and indicated by vertical-colored lines on traces in Fig. 3d) differs across dendritic locations. When ASAP3 signal was imaged at the soma, the moving bar arrived at the somatic ROI during the early phase of the depolarizing window of ASAP3 waveform regardless of the direction of the moving bar (Fig. 3d, position 0). In contrast, when ASAP3 signal was imaged at more distal dendritic locations, the relative timing between the arrival of the moving bar and ASAP3 signal in a given ROI differs between inward and outward motion directions (Fig. 3d, e, position bins 35, 65 and 85 μm, Fig. 3e). Towards the tip, ASAP3 imaging becomes more difficult due to low number of sensor molecules on thin dendrites, potentially leading to an underestimation of responses and decreased signal to noise ratio. However, the global signal is still detectable in distal dendrites. Beyond the inner third of SAC dendrites, the moving bar in the outward direction triggers synaptic inputs onto a local dendritic ROI near the peak of the ASAP3 waveform, but the bar moving in the inward direction triggers the synaptic inputs onto the same dendritic ROI when ASAP3 signal is still on the rising phase. These results provide direct experimental support for direction-dependent spatiotemporal dendritic integration in distal dendrites predicted by a previous computational modeling study^32^. In the outward motion direction, local glutamatergic inputs on the dendritic membrane show better coincidence with the global motion signal, which favors the initiation of suprathreshold calcium transients to trigger neurotransmitter release from distal varicosities.

### Emergence of direction-selective calcium responses in the middle segment of SAC dendrites

**Fig. 4:**
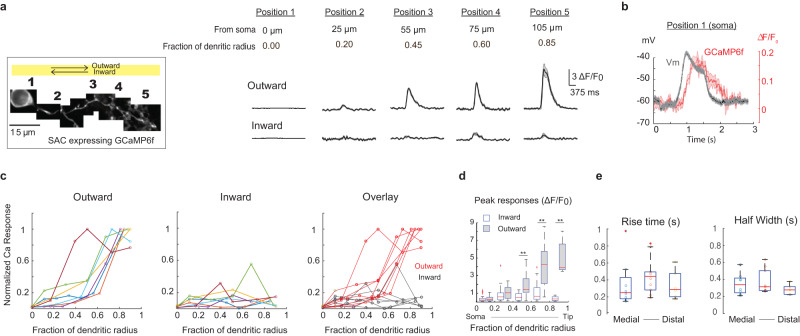
Direction-selectivity of calcium transients emerges in the distal half of SAC dendrites. **a** Left: ROIs (#1–5) of a SAC dendritic branch expressing GCaMP6f. Right: dF/F0 traces in ROIs on the left (Mean and SEM). **b** Overlay of Vm from current clamp recordings and GCaMP6f traces (Mean, SEM). **c** Normalized GCaMP6f responses at different distances from the soma along individual dendritic branches. Each line represents data from a single branch. The maximum response of each branch is normalized to 1. An abrupt increase of GCaMP6f signal is detected in distal half of the dendrite in the outward direction. **d** Binned dF/F0 and SEM of response amplitude at different fractions of dendritic radius. Kolmogorov-Smirnov Test, **p < 0.01, *n* = 11 cells. **e** Summary of rise time and half width of fast outward GCaMP6f transients in the distal half of dendrites. Bin size: 0.4–0.6; 0.6–0.8; 0.8–1. *n* = 11 cells. For each box in (**d**, **e**), the central mark indicates the median, and the bottom and top edges of the box indicate the 25th and 75th percentiles, respectively. The whiskers extend to the most extreme data points not considered outliers, and the outliers are plotted individually using the ‘+‘ symbol. Source data are provided as a Source Data file.

To study the transition from the isotropic motion response observed at the soma to the direction-selective response in distal SAC dendrites, we used two-photon imaging of GCaMP6f to monitor motion-evoked calcium transients at different subcellular locations of SACs (Fig. 4a). At the soma and the proximal half of the dendritic arbors, calcium signals are weak and not directionally tuned (Fig. 4a, c). Albeit weak, overlay of somatic Vm and the GCaMP6f fluorescence traces demonstrates that GCaMP6f can reliably detect a calcium increase at the soma (Fig. 4b, note different scale bars of GCaMP6f signal between Fig. 4a, b). Compared to the somatic Vm waveform, the somatic GCaMP6f waveform shows slower rise and decay times (Δ peak time: 162 ms; Δ decay time t1/2: 346 ms), at least partially due to the sensor kinetics^45^.

In contrast to the nondirectional, weak calcium response at the SAC proximal dendrites, calcium transients at the distal half of the dendritic arbor are directionally tuned to the outward motion direction (Fig. 4a, c). When we monitored calcium signals along SAC dendrites, we detected statistically significant differences between inward and outward responses in the distal half of the dendrites (Fig. 4d), consistent with a previous study by^7^. For outward motion direction, we observed an abrupt increase of calcium responses in the middle of each individual dendritic branch (Fig. 4c, outward). In contrast, the inward calcium responses along each dendritic branch remained weak throughout the dendrite (Fig. 4c, inward). The strong outward calcium transients share the same kinetics throughout the distal half of the dendritic branch (Fig. 4e), suggesting the dendritic initiation of suprathreshold calcium transients in the outward direction.

Together, voltage and calcium imaging experiments indicate that better coincidence of global and local dendritic activation in the outward motion direction leads to the initiation of strong suprathreshold calcium transients in the distal half of the SAC dendrites (Figs. 3 and 4).

### mGluR2 signaling is required for the direction-selective initiation of calcium transients in SAC dendrites

**Fig. 5:**
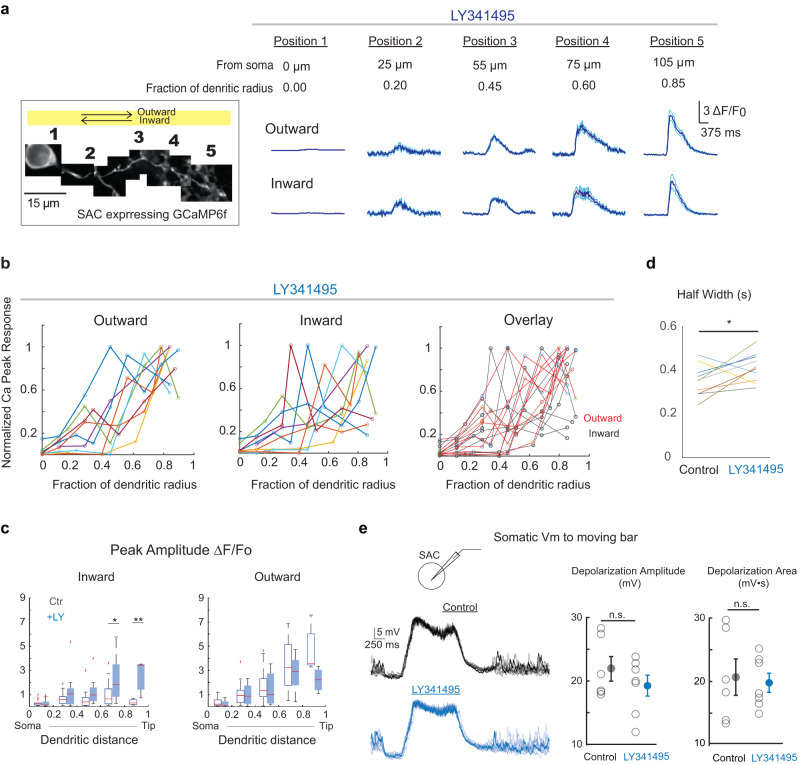
mGluR2 signaling is required to prevent the initiation of strong calcium transients in SAC dendrites during inward motion. **a** Same as Fig. 4a, but after applying LY341495. **b** As in Fig. 4c, normalized GCaMP6f responses at different distances from the soma along individual dendritic branches after applying LY341495. Each line represents data from a single branch. In LY341395, in the inward direction, large GCaMP6f responses are more frequently detected during imaging along the dendrite. **c** Summary of GCaMP6f responses (dF/F0) before and after LY341495 application. Control data is the same as in Fig. 4d; LY341395, *n* = 10 cells. Kolmogorov-Smirnov Test *p = 0.01, **p < 0.01. For each box, the central mark indicates the median, and the bottom and top edges of the box indicate the 25th and 75th percentiles, respectively. The whiskers extend to the most extreme data points not considered outliers, and the outliers are plotted individually using the ‘+‘ symbol. **d** Paired comparison of outward GCaMP6f transient half width in the distal half of the dendrites before and after applying LY341495. Two-tailed paired *t* test, *p = 0.03. *N* = 10 cells. **e** Left: Example Vm traces of current clamp recordings of SAC during the moving bar stimulus before and after LY341495. Traces are from individual trials. Darker traces are example single trials for easier visualization. Right: Summary plots of the amplitude of depolarization and the area of the trace during the depolarizing window. *N* = 6 cells for control, *N* = 7 cells for LY341485. Data are presented as mean values ± SEM. Two-sided unpaired *t* test, Bonferroni correction was made for multiple comparisons. *p* = 0.3 (left) and *p* = 0.8 (right). Source data are provided as a Source Data file.

**Fig. 6:**
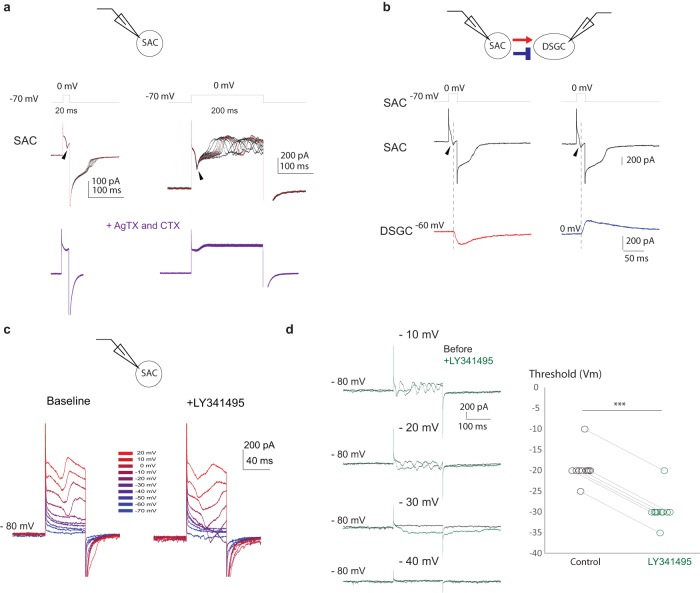
mGluR2 signaling increases the threshold of voltage-gated calcium channels in SACs. **a** Voltage clamp recordings of SACs. Currents evoked by short (left) and long (right) depolarizing steps were recorded before and after the application of N and P/Q type calcium channel antagonists ω-conotoxin (CTX) and ω-Agatoxin IVA (AgTX). Arrowheads indicate peaks of fast calcium currents. Regenerative calcium spikes are present during long depolarization (upper right traces) and fully blocked by Ca channel antagonists. Modified from Koren. et al., 2015, Fig. S4. **b** Example traces of paired SAC-DSGC voltage clamp recording from a null side SAC-DSGC pair. Dashed vertical lines indicate the relative time between the presynaptic SAC calcium currents and the postsynaptic DSGC EPSC (left) and IPSC (right). **c** Currents of a SAC in response to deploarizing voltage steps before and after application of LY341395. **d** Left: Currents of a SAC in response to different levels of 200 ms voltage steps before and after LY341495. Right: Vm threshold of calcium currents before and after LY341495. Lines connect the same cells before and after LY application. Two-sided paired student *t* test, *p* = 0.0002, *n* = 10 cells. Source data are provided as a Source Data file.

Direction-selective initiation of strong calcium transients in SAC dendrites implies a well-controlled calcium channel threshold that lies between the dendritic Vm depolarization levels evoked in inward and outward motion directions. To investigate the regulatory mechanism underlying the initiation of suprathreshold calcium transients, we focused on mGluR2 signaling in SACs because mGluR2 activation inhibits voltage-gated calcium channels of SACs and is required for the direction selectivity of SAC dendrites^12^. First, we examined the calcium response in distal SAC dendrites before and after application of the mGluR2 antagonist LY341495. Consistent with previous findings^12^, upon mGluR2 blockade, the direction selectivity of distal dendrites is impaired due to stronger inward motion response (Fig. 5a–c). To test whether the increased inward motion response results from the backpropagation of dendritic spikes from the opposite, outwardly activated dendritic sector, or results from the initiation of local suprathreshold calcium transients within the inwardly activated sector, we compared calcium signals from the soma to the distal dendrites before and during mGluR2 blockade. During mGluR2 blockade, strong calcium transients were not only observed in dendrites for outward motion direction, but were also more frequently observed in dendrites in the inward motion direction (Fig. 5a, b). The averaged inward responses in the outer half of SAC dendrites are statistically different before and after mGluR2 blockade (Fig. 5c, left panel). mGluR2 blockade does not affect the somatic calcium response amplitude in the inward direction. Somatic Vm waveforms are not altered by mGluR2 blockade in current clamp recordings for either direction (Fig. 5e). We also noted that distal calcium transients during mGluR2 blockade exhibit longer half width compared to the control condition (Fig. 5d), reflecting increased conductance of voltage-gated calcium channels upon mGluR2 blockade (see next section and Fig. 6). Therefore, mGluR2 blockade promotes the local initiation of suprathreshold calcium transients in dendrites without affecting the motion response at the soma.

### mGluR2 blockade lowers the threshold of voltage-gated calcium channels in SAC dendrites

Since the application of mGluR2 agonists inhibits voltage-gated calcium channels on SACs^12^, we hypothesize that blocking endogenous mGluR2 signaling reduces the calcium channel threshold as well as reduces the conductance of activated channels in SAC dendrites and thereby promotes suprathreshold calcium transients triggered by inward motion. To test this hypothesis, we recorded voltage-gated calcium currents in SACs in the voltage clamp mode with a Cs-based internal solution to block voltage-gated potassium channels intracellularly. A fast inward calcium current is evoked by a brief (20 ms) membrane depolarization (Fig. 6a, left, arrowheads) that triggers a precisely time-locked postsynaptic response in DSGCs in paired SAC-DSGC recordings (Fig. 6b). During prolonged (200 ms) depolarization, the calcium current shows a regenerative pattern resembling unclamped spikes likely due to inadequate space clamp in the SAC dendrites, which is fully blocked by Cd^12^ or a combination of N and P/Q type voltage-gated calcium channel antagonists^12^ (Fig. 6a, AgTX+CTX). Upon mGluR2 blockade, the calcium current was activated at the lower voltage threshold compared to that in the control condition (Fig. 6c, d), supporting a role of tonic mGluR2 signaling in modulating the calcium channel threshold in SAC dendrites. Together, these data indicate that decreased threshold of voltage-gated calcium channels underlies the emergence of suprathreshold calcium transients in inwardly activated dendrites shown in Fig. 5, thereby causing a decrease in dendritic direction selectivity.

### Kv3 channels stabilize SAC somatic motion responses

In contrast to direction-selective calcium transients in distal dendrites, the motion signal at the SAC soma is non-directional and low pass filtered. This low pass filtering effectively shields individual dendritic sectors from the influence of the directional tuning of other sectors. To study the mechanism underlying the low pass filtered global motion signal at the soma, we focused on the Kv3 channel, which has been reported to be enriched in the SAC perisomatic compartment and hypothesized to provide voltage-dependent shunting to isolate SAC dendritic sectors^36^. Here we examined the role of Kv3 signaling in SAC motion processing by measuring motion-evoked somatic response before and after Kv3 blockade.

**Fig. 7:**
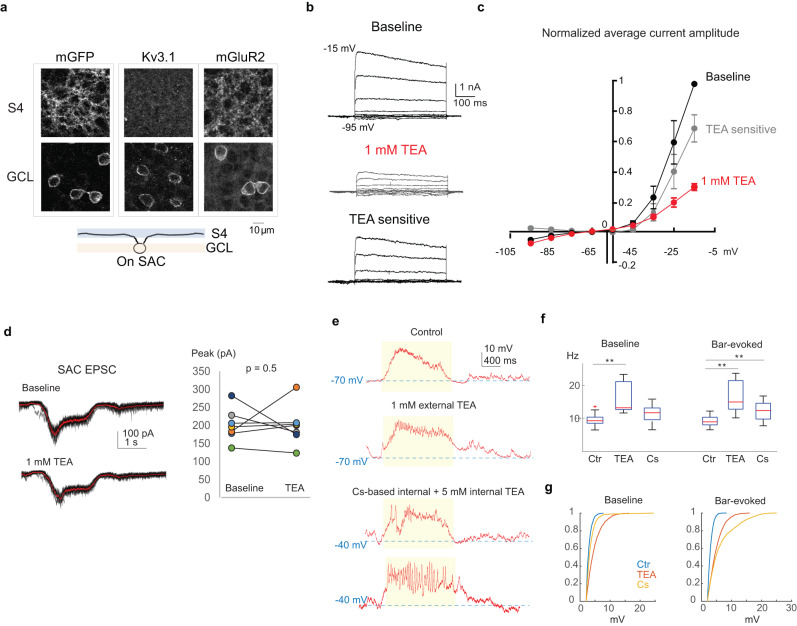
Kv3 channel prevents fast voltage fluctuations at SAC soma. **a** Fluorescence signals from SACs expressing a membrane-bound GFP (mGFP, left), or immunostained for Kv3.1 (middle) and mGluR2 (right) at the dendritic layer (S4, upper panels) and cell body layer (GCL, lower pannels). This experiment was repeated three times with similar results. **b** SAC currents at different voltage steps before and after applying 1 mM extracellular TEA (upper and middle), and isolated TEA-sensitive currents after subtraction between the two (lower). **c** I–V relationship of potassium current components, *n* = 6 cells. Data are presented as mean values ± SEM. **d** SAC EPSCs to flashing spot stimulus in control condition and in 1 mM TEA (black: individual trials; red: mean). Baseline: 304 + /− 47 pA; after TEA: 323 + /− 39 pA; *p* = 0.22, two-sided paired *t* test, *n* = 8 cells. **e** SAC somatic Vm during the moving bar stimulus under different conditions that block potassium channels. Yellow shaded area represents the period during which the bar is present in the receptive field. **f** Summary plot of the frequency of fast voltage events before (Baseline) and during moving bar stimulation (Bar-evoked) under conditions in (**e**). Control: *n* = 13 cells; TEA: 1 mM external TEA, *n* = 8 cells; Cs: Cs-based internal solution with 5 mM intracellular TEA, *n* = 11 cells. For each box, the central mark indicates the median, and the bottom and top edges of the box indicate the 25th and 75th percentiles, respectively. The whiskers extend to the most extreme data points not considered outliers, and the outliers are plotted individually using the ‘+‘ symbol. Kolmogorov-Smirnov Test, **p < 0.01. Bonferroni correction was made for multiple comparisons. **g** Cummulative distribution of peak amplitude of fast Vm events under conditions in (**e**). Source data are provided as a Source Data file.

First, we performed immunostaining of Kv3 and mGluR2 proteins in the mouse retina, and found that consistent with previous reports^36^, Kv3 expression is specifically localized around the soma, while mGluR2 expression is detectable in both the SAC soma and the dendritic plexus (Fig. 7a).

Next, we measured voltage-gated potassium currents in SACs by voltage clamp recording before and after applying 1 mM extracellular tetraethylammonium chloride (TEA). At this low concentration, TEA has been shown to be highly selective for blocking Kv3 channels but few other potassium channels^46^, and effectively blocks the delayed rectifier potassium current in mouse SACs^36^. Consistent with previous findings, we found that 1 mM TEA blocks the majority of the voltage-gated potassium conductance in SACs (Fig. 7b, c). We also found that excitatory synaptic inputs onto SACs in response to flashing spots are not significantly affected by 1 mM TEA application (Fig. 7d), consistent with lack of Kv3 gene expression in mouse bipolar cells^47^. Therefore, the effect of TEA treatment on SAC Vm is primarily attributed to altered SAC dendritic processing by Kv3 channel blockade rather than to changes in excitatory inputs.

Although the Kv3 channel-mediated conductance has been characterized in SACs^46^, the effect of blocking Kv3 channel in SAC light response has not been experimentally measured. Therefore, we recorded somatic Vm during the moving bar stimulus before and after bath applying 1 mM TEA. We found that Kv3 blockade does not significantly change the resting membrane potential (control: mean, −64.8 mV, SEM, 1.2, n, 17 cells; 1 mM external TEA: mean, −66.4 mV, SEM, 3.1, *n* = 7) or the low frequency component of SAC Vm depolarization triggered by the moving bar stimulus (control: mean, 19.9 mV, SEM, 0.9, n, 17 cells; 1 mM external TEA: mean, −21.2 mV, SEM, 1.8, *n* = 7). However, Kv3 blockade significantly increases the frequency and amplitude of fast voltage transients riding on top of the slow depolarization both at the baseline and during the moving bar stimulation (Fig. 7e–g, Vm variance: control 1.6 + /− 2.5, 6 cells; TEA: 4.3 + /− 1.5; 5 cells. **p = 0.006).

To further test the role of potassium channels in shaping SAC somatic responses, we performed a more complete blockade of potassium channels from the intracellular side using a Cs-based internal solution containing 5 mM TEA. Under this condition, we observed more frequent fast voltage transients, and the emergence of large transients of amplitudes >15 mV during the moving bar-evoked depolarization phase, and occasionally also during the baseline period. The resting membrane potential (mean, −36.7, SEM, 3.7, n, 12) is elevated compared to the control or external application of 1 mM TEA (ANOVA *p < 0.0001) likely due to the blockade of additional potassium channels besides Kv3.

In summary, perisomatic Kv3 channels are important for maintaining a steady level of membrane depolarization and prevent the emergence of fast voltage transients at the soma during visual motion processing by SAC dendrites.

### SAC compartmentalization is abolished by blocking both Kv3 and mGluR2

**Fig. 8:**
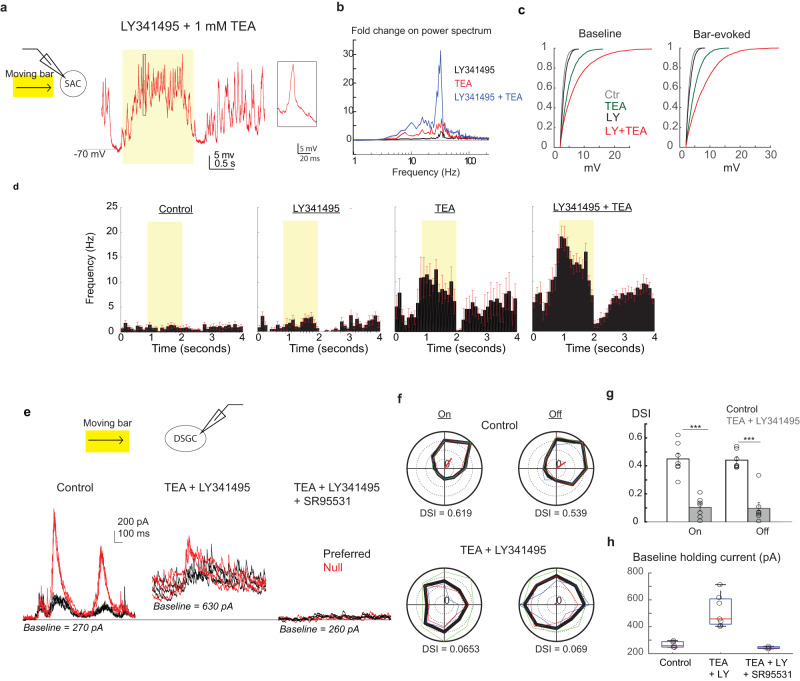
Blocking both Kv3 channel and mGluR2 unmasks large Vm transients at the SAC soma and abolishes the direction selectivity of DSGCs. **a** Example SAC Vm trace during the moving bar stimulus in the presence of LY341495 + 1 mM TEA. Inset shows an expanded view of a large-amplitude fast Vm event. Yellow shaded region corresponds to the timing of the moving bar passing through the SAC receptive field. **b** Power spectrum analysis under different conditions. Fold change is relative to control condition without antagonists. Control: *n* = 11 cells, LY341495: *n* = 8 cells, TEA: *n* = 7 cells, TEA + LY: *n* = 19 cells. **c** Cumulative distribution of peak amplitude of fast Vm events during the baseline period and during moving bar stimulation. **d** Peristimulus histograms (mean ± s.e.m) of fast voltage deflections detected a under different conditions. Yellow shaded regions correspond to the timing of the moving bar passing through the SAC receptive field. Control: *n* = 11 cells, LY341495: *n* = 8 cells, TEA: *n* = 7 cells, TEA + LY: *n* = 19 cells. **e** Example IPSCs from a Drd4-GFP+ On-Off DSGC under different conditions. Holding current at 0 mV is displayed under each condition. **f** Polar plots of On and Off components of IPSCs in (**e**). Colored lines correspond to individual trials and thick black line represents the average across 3 trials. Direction selectivity index (DSI) is shown under each polar plot. **g** Summary of DSI (mean ± s.e.m) of On and Off IPSC peak amplitude (*n* = 7 cells) before after application of TEA and LY341495 (red, On: DSI = 0.10 ± 0.03 and Off: DSI = 0.10 ± 0.04). ***p < 0.001. Bonferroni correction was made for multiple comparisons. **h** Summary of baseline holding current amplitude at 0 mV. Control, 5 cells; TEA + LY, 7 cells; TEA + LY + SR95531: 3 cells. Box plots are defined as in Fig. 2a. Source data are provided as a Source Data file.

In addition to blocking Kv3 or mGluR2 signaling individually, we also examined the effect of blocking both mechanisms by simultaneously applying the mGluR2 antagonist LY341495 and 1 mM extracellular TEA. We found that blockade of both Kv3 and mGluR2 causes a more drastic increase in the frequency and amplitude of fast voltage transients in SAC somatic Vm during both the baseline and the moving bar stimulation (Fig. 8a–d). As a result, direction-selective ganglion cells under this condition receive elevated tonic inhibitory currents that obscure light-evoked responses (Fig. 8e). Consequently, DSGC inhibitory postsynaptic currents (IPSCs) no longer exhibited directional tuning (Fig. 8e–h). Together, these results demonstrate the importance of the combined action of Kv3 and mGluR2 signaling in establishing the functional compartmentalization and the directional tuning of SAC dendrites.

## Discussion

### Concentrically organized dendritic mechanisms give rise to branch-specific directional tuning of SAC dendrites

The function of the SAC is conventionally described from the perspective of its output properties: synaptic release from each dendritic quadrant is direction-selective in the outward motion direction, and innervates one of the four DSGC subtypes that prefers upward, downward, anterior, or posterior direction (Fig. 1b). In this study, we investigated the architecture of SAC dendritic processing for integrating motion-evoked synaptic inputs. We found distinct forms of integration at the soma and dendrites. In dendrites, mGluR2 sets the appropriate calcium channel threshold for suprathreshold calcium transients in the outwardly activated dendrites. In contrast, SAC soma exhibits a low pass filtered global motion signal devoid of fast voltage or calcium transients. This global motion signal provides a steady depolarizing influence on individual dendrites that integrates with local synaptic inputs. Kv3 channels are required for minimizing fast voltage transients in the global motion signal, because Kv3 blockade causes an increase in fast voltage transients at the soma. When both mGluR2 and Kv3 signaling are blocked, frequent large spike-like transients appear at the SAC soma, and the directional tuning of DSGC inhibitory inputs is abolished due to hyperactive SACs before and during the motion stimulus. Together, concentrically organized dendritic machineries transform motion-evoked synaptic inputs into branch-specific outputs in distal dendrites. Our study thus highlights the importance of voltage-dependent dendritic mechanisms and their modulation by metabotropic glutamate receptors, which complement previously reported passive dendritic and synaptic mechanisms^7,8,13,22,23,26,28,29^, in setting up SAC dendritic direction selectivity.

### The timing of the global motion signal influences local synaptic integration at individual sectors

SAC dendritic quadrants exhibit independent directional preference and synapse onto distinct On-Off DSGC subtypes^10,20^. In contrast to quadrant-specific output properties, synaptic inputs do not show quadrant-specific clustering, but are instead radially distributed in concentric zones around the soma^7,8^. In previous models of SAC dendritic direction selectivity, much focus has been given to the dendritic integration of local synaptic inputs within a sector from soma to tip. However, the influence from other sectors to a given sector during dendritic motion processing is often overlooked, although it is evident that at least in proximal dendrites, motion-evoked responses should be contributed by synaptic inputs from all sectors. Here, our voltage imaging and patch clamp recording experiments demonstrate that the entire dendritic arbor of the SAC is subject to the electrotonic spread of subthreshold Vm depolarization as a result of the global integration of PSPs. This global motion signal is funneled and stabilized at the soma and spreads into the dendritic sectors, where it integrates with local synaptic inputs. The relative timing of local synaptic inputs and the global Vm signal in a given sector differs between the inward and outward directions. A better coincidence of global and local signals in the outwardly activated dendrite favors the distal dendritic Vm to rise above the calcium channel threshold, leading to suprathreshold calcium transients that trigger strong neurotransmitter release in the outward direction.

### mGluR2 signaling prevents the emergence of suprathreshold calcium transients in inwardly activated SAC dendrites

mGluR2 is selectively expressed in SACs in the retina^48–50^. As a Class II group mGluR, mGluR2 activation inhibits adenylyl cyclase through Gi/Go proteins to directly modulate ion channel functions^51^. Previous work from our group shows that in SACs, application of an mGluR2 agonist inhibits voltage-gated N- and P/Q-types of calcium channels, but not potassium channels^12^. Furthermore, blocking endogenous mGluR2 signaling by an antagonist causes increased inward motion response in the distal dendrites, and thereby reducing the direction selectivity of dendritic sectors^12^. When the moving stimulus is restricted to activate one side of the SAC dendritic field, mGluR2 blockade increases the likelihood of strong calcium responses in the distal dendrites on the opposite side^12^. These previous findings indicate an important role of endogenous mGluR2 signaling in maintaining compartmentalized signaling of SAC dendrites. However, how mGluR2-mediate calcium channel inhibition impacts dendritic integration has not been studied.

Our current study fills this knowledge gap by providing a mechanistic explanation of previously reported effect of mGluR2 blockade on SAC dendritic direction selectivity^12^. Two possible mechanisms could underlie the effect of mGluR2 blockade and the resulting disinhibition of calcium channel activity: (1) aberrant backpropagation of calcium spikes from the outward-activated sector to the opposite sector through the soma, which was postulated in our previous study^12^, or (2) A lower local dendritic spike threshold so that the global Vm depolarization more likely elicits dendritic spiking in the inwardly activated dendrites or in dendrites without local synaptic inputs. Our results in this study support the latter scenario. Because of the voltage-dependent shunting of the soma by Kv3 channels, somatic Vm and calcium responses are not significantly affected by mGluR2 blockade and no backpropagating spikes appear at the soma upon mGluR2 blockade, ruling out the first scenario. Instead, distal dendrites become more excitable upon mGluR2 blockade due to a lower calcium channel threshold, resulting in the emergence of suprathreshold calcium transients during inward motion stimulation.

**Fig. 9:**
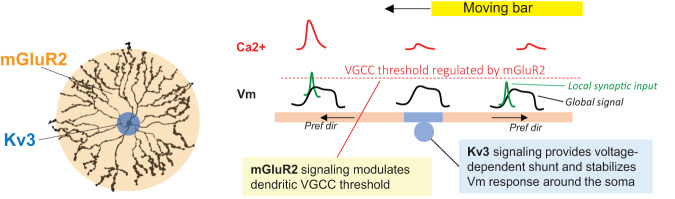
The concentric architecture of SAC dendritic processing units. Left: Kv3 and mGluR2 signaling impact perisomatic and dendritic zones respectively. Right: A model of mGluR2 and Kv3 signaling in generating outward direction selectivity of SAC dendrites.

In this study, we find that endogenous mGluR2 signaling in the dendrites and Kv3 signaling in the perisomatic region collectively implement compartmentalized signaling during visual motion integration across SAC dendrites. Our data show that mGluR2 signaling exerts its influence on VGCCs in dendrites, but does not alter the visual response around the soma because of the shunting action of perisomatic Kv3 channels. This arrangement partitions the dendritic arbor into two concentric computational subunits with different integration patterns. We use these findings to generate an updated mechanistic model of mGluR2 signaling in SAC direction selectivity that aids the re-interpretation of previous results with mGluR2 antagonists^12^ (Fig. 9).

The present results on mGluR2’s role in regulating dendritic calcium channels raise the interesting possibility that endogenous mGluR2 signaling may function as a homeostatic regulator of dendritic excitability in response to synaptic activity. When glutamate release from bipolar cells is high, such as during high contrast motion stimulation or noisy backgrounds, mGluR2 signaling might be upregulated to prevent excessive excitability of SAC dendrites that could otherwise cause excessive inward-direction calcium responses. Further studies are required to determine if activity-dependent mGluR2 signaling can fine-tune the dendritic calcium spike threshold and thereby extend the operating range of SAC dendritic direction selectivity.

### An unconventional role of Kv3 channels in the nonspiking dendritic compartment

Kv3.1/3.2 family delayed rectifier channels mediate rapidly activating and deactivating currents with little inactivation. They are key components in fast spiking neurons throughout the brain since these channels can rapidly repolarize cells after spiking^52^. Intriguingly, previous studies show that these channels mediate the majority of the SAC’s potassium currents, and are enriched in the SAC soma and proximal dendrites^36,53^, suggesting that these channels also play an important role in non-spiking subcellular compartments. Our results demonstrate that perisomatic Kv3 channels are activated by accumulating bipolar cell inputs triggered by the moving stimulus. Kv3 signaling clamps somatic Vm responses to minimize fast Vm transients due to its voltage-dependent shunting action. Therefore, Kv3 channels can stabilized Vm signal in non-spiking dendritic compartment, in addition to its signature role of spike repolarization in fast spiking neurons^52^.

Together, our study shows a concentric organization of SAC dendritic integration units underlying the independent direction selectivity of dendritic quadrants. When motion-evoked synaptic inputs arrive at SAC dendrites, mGluR2 signaling and Kv3 channels exert their influence in dendritic and perisomatic regions respectively to shape the spatiotemporal integration of motion signal. Metabotropic modulation and voltage-dependent membrane conductances are fundamental building blocks of neuronal signaling, but their roles in dendritic processing are less understood. Exploring algorithmic operations of these mechanisms in SAC dendrites may provide insights into understanding and predicting the computational power of dendrites in diverse neural circuits.

## Methods

### Mice

*Chat-IRES-Cre* mice (129S6-*Chat*^*tm2(cre)Lowl*^/J), *Chat-CreERT* (Chat^tm1(cre/ERT)Nat^) and floxed *tdTomato* mice (129S6-*Gt(ROSA)26Sor*^*tm9(CAG-tdTomato)Hze*^/J) were acquired from the Jackson Laboratory. *mGluR2–GFP* mouse line was a generous gift from Dr. Marla Feller at University of California, Berkeley. *Drd4–GFP* mice, in which posterior-preferring DSGCs are selectively labeled, were originally developed by MMRRC in the Swiss Webster background, and were subsequently backcrossed to C57BL/6 background. All strains were backcrossed to the *C57BL/6* background in our laboratory and crossed to each other to create the lines used in this study. For SAC recordings*, Chat-IRES-Cre/floxed tdTomato* double transgenic mice were used. For calcium imaging, *Chat-IRES-Cre* mice were used for AAV-floxed GCaMP6f expression in SACs. For ASAP imaging, we used *Chat-IRES-Cre* mice infected by AAV-floxed ASAP3 vectors for sparse labeling of SACs with ASAP3.

All experiments were performed on healthy mice of normal immune status which had not been previously used for tests or procedures. Mice were housed in 12 h-12 h light-dark cycles in groups of 2–5 per cage and were provided with food and water *ad libitum* at room temperature and 40–60% humidity. Mice of ages P21-P40 of both sexes were used, and data from sexes are first examined separately and then combined after no sex-specific difference was found. All procedures to maintain and use mice were in accordance with the University of Chicago Institutional Animal Care and Use Committee (Protocol number ACUP 72247) and in conformance with the NIH Guide for the Care and Use of Laboratory Animals and the Public Health Service Policy.

### Whole-mount retina preparation

Mice were anaesthetized with isoflurane and decapitated after dark adaptation. Under infrared illumination, retinas were isolated from the pigment epithelium at room temperature in oxygenated Ames’ medium (Sigma-Aldrich, St. Louis, MO). Isolated retinas were then cut into dorsal or ventral halves (Wei et al., 2010) and mounted ganglion-cell-layer-up on top of a 1 mm^2^ hole in a small piece of filter paper (Millipore, Billerica, MA). Retinas were kept in darkness at room temperature in Ames’ medium bubbled with 95% O2/5% CO^2^ until use (0–7 h).

### Visual stimulation

A white organic light-emitting display (OLEDXL, eMagin, Bellevue, WA; 800 × 600 pixel resolution, 60 Hz refresh rate) was controlled by an Intel Core Duo computer with a Windows 7 operating system and was presented to the retina at a resolution of 1.1 μm/pixel. Moving bar stimuli were generated by MATLAB2012 and the PsychToolbox (Brainard, 1997), and projected through the condenser lens of the two-photon microscope onto the photoreceptor layer. In the plane of the retina, the OLED image was centered on the soma of SAC of interest. For electrophysiology experiments, a positive-contrast bar (275 µm wide, 440 µm long) moved along the long axis at a speed of 330, 440, or 550 µm/s over a 660µm-diameter field on the retina; and three to five trials were recorded. For calcium and voltage imaging experiments, moving bars were presented in 10 trials of two pseudorandomized directions (centripetal and centrifugal), which were determined by dendrite orientation. These bars had dimensions of 275 μm × 220 μm. The speed of the bar was 440 μm/s for calcium imaging or 550 μm/s for voltage imaging. Unless otherwise noted, the intensity of the moving bar was ~6.3 × 10^4^ isomerizations (R*)/rod/s, lying in the photopic range, and the background intensity was ~1800 R*/rod/s, lying at the lower end of the photopic range.

### Two-photon calcium imaging of GCaMP6f fluorescence in SACs

Genetically encoded calcium indicator GCaMP6f was expressed in sparse populations of On and Off SACs by intravitreal injection of an 7m8 modified AAV vector carrying CAG-promoter driven floxed GCaMP6f (gift from the Dr. Sui Wang at Stanford University) into *Chat-IRES-Cre* mice aged P14-25. Mice were anesthetized using 4 μL/g intraperitoneal injection of 10% ketamine/5% xylazine in PBS. Animals were placed on their side and the periorbital region was locally anesthetized using one drop of 0.5% proparacaine HCl (Henry Schein, Melville, NY). Using a Femtojet 4i microinjector (Eppendorf, Hamburg, Germany) equipped with sharp pulled glass-pipettes, 1–1.5 µL of AAV vector solution was injected into the vitreal space of both eyes. Retinas were isolated, screened, and imaged at 4–8 weeks following injection.

GCaMP6 fluorescence of isolated retinas in oxygenated Ames at 32–33 °C was imaged in a customized two-photon laser scanning fluorescence microscope (Bruker Nano Surfaces Division). GCaMP6 was excited by a Ti:sapphire laser (Coherent, Chameleon Ultra II, Santa Clara, CA) tuned to 920 nm, and the laser power was adjusted to avoid saturation of the fluorescent signal. Onset of laser scanning induces a transient On response in On SACs that adapts to the baseline in ~3 s. Therefore, to ensure the complete adaptation of this laser-induced response and a stable baseline, visual stimuli were given after 10 s of continuous laser scanning. To separate the visual stimulus from GCaMP6 fluorescence, a band-pass filter (Semrock, Rochester, MA) was placed on the OLED to pass blue light peaked at 470 nm, while two notched filters (Bruker Nano Surfaces Division) were placed before the photomultiplier tubes to block light of the same wavelength. Imaging was performed in the regions of the retina that contain sparsely labeled SACs so that individual dendrites and varicosities could be resolved and the orientation of the dendrites relative to the soma could be determined. A water immersion objective (60x, Olympus LUMPlanFl/IR) was used. Time series of fluorescence were collected at 20–50 Hz.

### Calcium imaging analysis

Analysis was performed using Fiji/ImageJ 1.53c and MATLAB2021b. Regions of interest (ROIs) corresponding to individual varicosities, dendritic shafts, soma, and background were manually selected in ImageJ. The fluorescent time course of each ROI was determined by averaging all pixels within the ROI for each frame. The fluorescence of the background region was subtracted from the raw fluorescent signals of the ROIs in the same imaging window at each time frame. The moving bar visual stimulus used for calcium imaging included 500 ms interval between the end of one sweep and the start of another. Fluorescence intensities during these intersweep intervals were used to create a baseline (F0) trace for each ROI by fitting a two-term exponential decay function. Fluorescence measurements were then converted to ΔF/F0 values by calculating ΔF = (F − F0)/F0 for every datapoint. Trials for given ROIs were discarded if baseline fluorescence values dropped below 0.1 since this could lead to arbitrarily large visual evoked responses. Remaining samples were then resampled to 75 Hz through boxcar method and smoothed using an average sliding window of 4 datapoints. ΔF/F0 traces were then clipped, sorted by visual stimulus direction (inward or outward), and averaged over 5–10 trials. Prior to further analysis, ROIs were subjected to a response quality test QI = Var[Avg. Resp]/Avg(Var[R(t)]) ≥ 0.45 to ensure consistency across trials^54^. Peak and area ΔF/F0 values, along with time to peak and rise slope for centripetal versus centrifugal responses were calculated using custom MATLAB scripts. Direction selectivity index (DSI), defined as (ΔF/F0_outward - ΔF/F0_inward)/(ΔF/F0_outward + ΔF/F0_inward), was calculated for all ROIs. Lastly, the dendritic fraction for each ROI was calculated by (distance soma to ROI)/(distance soma to dendritic tip).

### Two-photon voltage imaging of ASAP family genetically encoded voltage indicators (GEVIs) in SACs

We utilized ASAP3 (gift from Dr. Michael Lin, Stanford University) for voltage imaging of SACs. ASAP3 was expressed the same way as GCaMP6f (AAV intravitreal injection into ChAT-Cre mice). Retinas from AAV-floxed ASAP3 injected mice were isolated 5 weeks post injection. Two-photon imaging was performed similarly as GCaMP6 with the exception that imaging was performed at 250-300 Hz.

### Voltage imaging analysis

ASAP fluorescence traces of ROIs were acquired with similar methods used for calcium imaging analysis (see the previous section). The traces were then resampled to 1000 Hz through boxcar method and smoothed using an average sliding window of 15 datapoints. ΔF/F0 traces were then clipped, sorted by visual stimulus direction (inward or outward), and averaged over 10 trials. Prior to further analysis, traces were subjected to the same response quality test as for calcium imaging with QI ≥ 0.15 to ensure consistency across trials. Changes ASAP3 signals were converted to changes in Vm values according to the previously published and measured fluorescence-voltage relationships^42,43^. The relative timing of local synaptic inputs in each ROI was estimated according to Fig. 2b, c. For timing analysis, the depolarizing and hyperpolarizing events are identified as the trace reaches above or below 2 SD of the mean baseline level for a minimal period of 100 ms after the onset of the stimulus.

### Cell-targeted patch-clamp electrophysiology

Recordings in this study were performed from On SACs and posterior-preferring DSGCs. Cells were visualized with infrared light (920 nm) and an IR-sensitive video camera (Watec). For current-clamp recordings, SACs were targeted using *Chat-IRES-Cre*/floxed *tdTomato* mice. Cell identity was confirmed by morphology and physiologically by the lack of action potentials from the voltage traces. Recording electrodes of 3.5–4.5 MΩ were backfilled with a potassium-based internal solution containing (from Sigma): 120 mM KMeSO4, 10 mM KCl, 0.01 mM CaCl⋅2H_2_O, 10 mM HEPES, 0.1 mM EGTA, 2 mM adenosine 5′-triphosphate (magnesium salt), 0.4 mM guanosine 5′-triphosphate (trisodium salt), 10 mM phosphocreatine (disodium salt),and pH was balanced to 7.25 with KOH. For a subset of experiments, a Cs-based internal solution is used for Vm recordings (in mM): 110 CsMeSO3, 2.8 NaCl, 4 EGTA, 5 TEA-Cl, 4 adenosine 5’- triphosphate (magnesium salt), 0.3 guanosine 5’-triphosphate (trisodium salt), 20 HEPES, 10 phosphocreatine (disodium salt), and CsOH to pH 7.2. Electrophysiological recordings were acquired at 10 kHz with a Multiclamp 700 A amplifier (Molecular Devices) using pCLAMP 10.4 recording software and a Digidata 1440 digitizer. Membrane potential was monitored in current-clamp mode (holding current = 0 pA) during moving bar visual stimulation. For a subset of experiments, SACs currents were monitored using K+ internal in voltage-clamp mode while providing 500 ms voltage steps ranging from −95 mV to +45 mV. In voltage-clamp mode, whole-cell and series resistance (70% at 5 kHz) compensation were performed.

For recordings of synaptic currents, posterior-preferring DSGCs were targeted using Drd4-GFP mice. Recording electrodes were filled with an internal solution containing (in mM): 110 CsMeSO3, 2.8 NaCl, 4 EGTA, 5 TEA-Cl, 4 adenosine 5’- triphosphate (magnesium salt), 0.3 guanosine 5’-triphosphate (trisodium salt), 20 HEPES, 10 phosphocreatine (disodium salt), and CsOH to pH 7.2. Currents were recorded at a holding potential of 0 mV while presenting moving bar visual stimulation.

### Data analysis for electrophysiological recordings

Current-clamp and voltage-clamp data were analyzed using a combination of Molecular Devices Clampfit software and custom protocols in MATLAB. Depolarization amplitudes and area were computed from the low-frequency component of the voltage recording in MATLAB. To isolate the low-frequency component from the recorded voltage traces, we took the average across 5 trials and passed this average trace through a low-pass filter with a cutoff frequency of 20 Hz. Then local minima were identified between 0 and 0.5 s time window to calculate a baseline Vm value. Finally, the depolarization amplitude was defined as the difference between maximum Vm (between 0.5 s and 2 s) and baseline Vm and depolarization area was defined as the area between the Vm trace (0.5 s to 2 s) and the baseline Vm. Power spectra were computed in MATLAB for individual trials across all voltage recordings for frequencies ranging from 0 to 2,500, without low pass filtering. We then calculated the average power spectrum for each testing condition (baseline, LY341495, TEA, LY & TEA) and the fold change from base across all frequencies for each of the drug conditions. For analysis of the high-frequency fast voltage deflections during SAC Vm recording, local minima were identified every 50 ms along each individual trial to create a rolling adjusting baseline representative of the low-frequency depolarization component. We then fragmented the individual traces into these 50 ms segments and searched for voltage peaks that were 4 mV or more above the baseline for that segment, had peak prominence of 2 mV or more, had widths at half-prominence between 4 and 50 ms, and were at least 15 ms apart. For each peak detected, amplitude (peak – baseline), prominence, time of peak, rise time (10% to 90%), decay time (90% to 10%), and width at half prominence were measured. From these, we calculated and plotted peri-stimulus time histograms. Inhibitory postsynaptic currents (IPSCs) were recorded from DSGCs in response to a moving bar stimulus. IPSC amplitude, charge transfer, and baseline holding currents were measured in Clampfit software for both On (leading edge) and Off (trailing edge) components across 3 trials of 8 directions of the moving bar stimulus. The average amplitude and charge transfer for all directions were calculated across the 3 trials. Direction selectivity index was calculated using the average responses in the null (maximal IPSC response) and preferred (opposite to null) directions as follows: *DSI*= *Rnull*– *Rpref*/*Rnull*+ *Rpref*. Calcium current analysis in Fig. 6: Inward currents in response to depolarization steps in Cs-based internal were measured by first fitting the first 1–6 ms during the voltage step with an exponential function, and then subtracting the measured current from the fitted curve. Calcium current threshold is determined by the first depolarizing voltage step that triggers an inward current with an amplitude >2 SD of the baseline fluctuations and confirmed by visual inspection.

### Immunohistochemistry

Retinas were fixed in paraformaldehyde (PFA, 4% in PBS) for 20 min on a 24-well plate. Following fixation, the 24-well plate was plate on a shaker and the retina pieces were washed in PBS three times for 20 min. The retina pieces were incubated in block solution (10% donkey serum, 1% BSA and 0.5% TritonX in PBS) 3X for 20 min. The retina pieces were incubated with one or more of the following primary antibodies for 72 h at 4 C: Abcam #Ab238432: Rabbit monoclonal anti-mGluR2 antibody (1:500) and Invitrogen #MA5-27684 Mouse monoclonal anti-Kv3.1 antibody (1:1000). After primary antibody incubation, the retina pieces were rinsed with block solution 3X for 20 min on the shaker at room temperature. The retina tissues were then incubated Alexa-conjugate secondary antibodies Alexa-488 conjugated goat anti-mouse (Invitrogen Cat # A32723) and Alexa-594 conjugated donkey anti-rabbit (Invitrogen Cat # A-21207) at 1:750 or 1:1000 concentrations for 5 h. Following secondary antibody incubation, the retina tissues were rinsed in PBS 3X for 20 min. The retina pieces were left in the last PBS wash for 2 h before mounting. The retina tissues were then mounted onto glass slides using Vectashield and imaged using a two-photon microscope.

### Statistical analysis

Sample size in each group was calculated based on preliminary data to have a power of test stronger than 0.8. Grouped data with Gaussian distribution were presented as mean ± SEM in summary graphs with scattered dots representing individual cells. Two-sided two-sample paired or unpaired *t* test was used to compare two sample groups. Grouped data with non-Gaussian distribution were presented as box plots and tested with Kolmogorov-Smirnov test or Wilcoxon Rank Sum test. For multiple comparisons, *p* values were adjusted with false discovery rate (FDR) correction^55^. *P* < 0.05 was considered significant; n.s. stands for no significance; *p < 0.05; **p < 0.01; ***p < 0.001 unless otherwise indicated. Sample sizes were indicated in figure legends. For box plots, the central mark indicates the median, and the bottom and top edges of the box indicate the 25th and 75th percentiles, respectively. The whiskers extend to the most extreme data points not considered outliers, and the outliers are plotted individually using the ‘+‘ marker symbol.

### Reporting summary

Further information on research design is available in the Nature Portfolio Reporting Summary linked to this article.

### Supplementary information

Reporting Summary

### Source data

Source Data

## Data Availability

Source data are provided with this paper.

## Code availability

Custom codes are available at https://github.com/ww-uchicago/NC-manuscript-2023.

## References

[1] Branco T, Häusser M (2010). The single dendritic branch as a fundamental functional unit in the nervous system. Curr. Opin. Neurobiol..

[2] Stuart GJ, Spruston N (2015). Dendritic integration: 60 years of progress. Nat. Neurosci..

[3] Tran-Van-Minh A (2015). Contribution of sublinear and supralinear dendritic integration to neuronal computations. Front. Cell. Neurosci..

[4] Brecha N, Johnson D, Peichl L, Wässle H (1988). Cholinergic amacrine cells of the rabbit retina contain glutamate decarboxylase and gamma-aminobutyrate immunoreactivity. Proc. Natl. Acad. Sci. USA..

[5] Famiglietti EV (1991). Synaptic organization of starburst amacrine cells in rabbit retina: analysis of serial thin sections by electron microscopy and graphic reconstruction. J. Comp. Neurol..

[6] Vaney DI, Young HM (1988). GABA-like immunoreactivity in cholinergic amacrine cells of the rabbit retina. Brain Res..

[7] Vlasits ALL (2016). A role for synaptic input distribution in a dendritic computation of motion direction in the retina. Neuron.

[8] Ding H, Smith RG, Poleg-Polsky A, Diamond JS, Briggman KL (2016). Species-specific wiring for direction selectivity in the mammalian retina. Nature.

[9] Pottackal J, Singer JH, Demb JB (2021). Computational and molecular properties of starburst amacrine cell synapses differ with postsynaptic cell type. Front. Cell. Neurosci..

[10] Euler T, Detwiler PB, Denk W (2002). Directionally selective calcium signals in dendrites of starburst amacrine cells. Nature.

[11] Chen Q, Pei Z, Koren D, Wei W (2016). Stimulus-dependent recruitment of lateral inhibition underlies retinal direction selectivity. Elife.

[12] Koren D, Grove JCR, Wei W (2017). Cross-compartmental modulation of dendritic signals for retinal direction selectivity. Neuron.

[13] Poleg-Polsky A, Ding H, Diamond JS (2018). Functional compartmentalization within starburst amacrine cell dendrites in the retina. Cell Rep..

[14] Vaney DI, Sivyer B, Taylor WR (2012). Direction selectivity in the retina: symmetry and asymmetry in structure and function. Nat. Rev. Neurosci..

[15] Oyster CW, Barlow HB, Takahashi E (1967). Direction-selective units in rabbit retina: distribution of preferred directions. Science.

[16] Sabbah S (2017). A retinal code for motion along the gravitational and body axes. Nature.

[17] Lee S, Kim K, Zhou ZJ (2010). Role of ACh-GABA cotransmission in detecting image motion and motion direction. Neuron.

[18] Wei W, Hamby AM, Zhou K, Feller MB (2011). Development of asymmetric inhibition underlying direction selectivity in the retina. Nature.

[19] Yonehara K (2011). Spatially asymmetric reorganization of inhibition establishes a motion-sensitive circuit. Nature.

[20] Briggman KL, Helmstaedter M, Denk W (2011). Wiring specificity in the direction-selectivity circuit of the retina. Nature.

[21] Fried SI, Münch TA, Werblin FS (2002). Mechanisms and circuitry underlying directional selectivity in the retina. Nature.

[22] Fransen JW, Borghuis BG (2017). Temporally diverse excitation generates direction-selective responses in ON- and OFF-type retinal starburst amacrine cells. Cell Rep..

[23] Kim JS (2014). Space-time wiring specificity supports direction selectivity in the retina. Nature.

[24] Trenholm S, McLaughlin AJ, Schwab DJ, Awatramani GB (2013). Dynamic tuning of electrical and chemical synaptic transmission in a network of motion coding retinal neurons. J. Neurosci..

[25] Srivastava P (2022). Spatiotemporal properties of glutamate input support direction selectivity in the dendrites of retinal starburst amacrine cells. Elife.

[26] Stincic T, Smith RG, Taylor WR (2016). Time course of EPSCs in ON-type starburst amacrine cells is independent of dendritic location. J. Physiol..

[27] Gaynes, J. A., Budoff, S. A., Grybko, M. J. & Poleg-Polsky, A. Heterogeneous presynaptic receptive fields contribute to directional tuning in starburst amacrine cells. (2023). 10.7554/elife.90456.1 10.7554/eLife.90456 PMC10752589 38149980

[28] Strauss S (2022). Center-surround interactions underlie bipolar cell motion sensitivity in the mouse retina. Nat. Commun..

[29] Gaynes JA, Budoff SA, Grybko MJ, Hunt JB, Poleg-Polsky A (2022). Classical center-surround receptive fields facilitate novel object detection in retinal bipolar cells. Nat. Commun..

[30] Lee S, Zhou ZJ (2006). The synaptic mechanism of direction selectivity in distal processes of starburst amacrine cells. Neuron.

[31] Kostadinov D, Sanes JR (2015). Protocadherin-dependent dendritic self-avoidance regulates neural connectivity and circuit function. Elife.

[32] Tukker JJ, Taylor WR, Smith RG (2004). Direction selectivity in a model of the starburst amacrine cell. Vis. Neurosci..

[33] Miller RF, Bloomfield SA (1983). Electroanatomy of a unique amacrine cell in the rabbit retina. Proc. Natl. Acad. Sci. USA.

[34] Hausselt SE, Euler T, Detwiler PB, Denk W (2007). A dendrite-autonomous mechanism for direction selectivity in retinal starburst amacrine cells. PLoS Biol..

[35] Oesch NW, Taylor WR (2010). Tetrodotoxin-resistant sodium channels contribute to directional responses in starburst amacrine cells. PLoS One.

[36] Ozaita A (2004). A unique role for Kv3 voltage-gated potassium channels in starburst amacrine cell signaling in mouse retina. J. Neurosci..

[37] Xu H-P (2016). Retinal wave patterns are governed by mutual excitation among starburst amacrine cells and drive the refinement and maintenance of visual circuits. J. Neurosci..

[38] Chen Q, Smith RG, Huang X, Wei W (2020). Preserving inhibition with a disinhibitory microcircuit in the retina. Elife.

[39] Helmstaedter M (2013). Connectomic reconstruction of the inner plexiform layer in the mouse retina. Nature.

[40] Franke K (2017). Inhibition decorrelates visual feature representations in the inner retina. Nature.

[41] Berntson A, Taylor WR (2000). Response characteristics and receptive field widths of on-bipolar cells in the mouse retina. J. Physiol..

[42] Villette V (2019). Ultrafast two-photon imaging of a high-gain voltage indicator in awake behaving mice. Cell.

[43] Chamberland S (2017). Fast two-photon imaging of subcellular voltage dynamics in neuronal tissue with genetically encoded indicators. Elife.

[44] Yang HH (2016). Subcellular imaging of voltage and calcium signals reveals neural processing in vivo. Cell.

[45] Chen T-W (2013). Ultrasensitive fluorescent proteins for imaging neuronal activity. Nature.

[46] RUDY B (1999). Contributions of Kv3 Channels to Neuronal Excitability. Ann. N. Y. Acad. Sci..

[47] Shekhar K (2016). Comprehensive classification of retinal bipolar neurons by single-cell transcriptomics. Cell.

[48] Cai W, Pourcho RG (1999). Localization of metabotropic glutamate receptors mGluR1alpha and mGluR2/3 in the cat retina.. J. Comp. Neurol..

[49] Seebahn A, Rose M, Enz R (2008). RanBPM is expressed in synaptic layers of the mammalian retina and binds to metabotropic glutamate receptors. FEBS Lett..

[50] Koulen P, Malitschek B, Kuhn R, Wässle H, Brandstätter JH (1996). III metabotropic glutamate receptors in the rat retina: distributions and developmental expression patterns. Eur. J. Neurosci..

[51] Niswender CM, Conn PJ (2010). Metabotropic glutamate receptors: physiology, pharmacology, and disease. Annu. Rev. Pharmacol. Toxicol..

[52] Rudy B, McBain CJ (2001). Kv3 channels: voltage-gated K^+^ channels designed for high-frequency repetitive firing. Trends Neurosci..

[53] Kaneda M, Ito K, Morishima Y, Shigematsu Y, Shimoda Y (2007). Characterization of voltage-gated ionic channels in cholinergic amacrine cells in the mouse retina. J. Neurophysiol..

[54] Baden T (2016). The functional diversity of retinal ganglion cells in the mouse. Nature.

[55] Jafari M, Ansari-Pour N (2019). Why, when and how to adjust your p values?. Cell J..

