# Supplementary material for: Dendritic mGluR2 and perisomatic Kv3 signaling regulate dendritic computation of mouse starburst amacrine cells
Source: Nat Commun. 2024 Feb 28;15:1819. doi: 10.1038/s41467-024-46234-7 (PMC10901804; doi:10.1038/s41467-024-46234-7)
Supplement: Supplementary file 1 — Reporting Summary [file 41467_2024_46234_MOESM1_ESM.pdf]

nature portfolio

Corresponding author(s): Wei Wei

Last updated by author(s): Jan 24, 2024

Reporting Summary

Nature Portfolio wishes to improve the reproducibility of the work that we publish. This form provides structure for consistency and transparency in reporting. For further information on Nature Portfolio policies, see our Editorial Policies and the Editorial Policy Checklist.

Statistics

For all statistical analyses, confirm that the following items are present in the figure legend, table legend, main text, or Methods section.

n/a	Confirmed	
☐	☒	The exact sample size (*n*) for each experimental group/condition, given as a discrete number and unit of measurement
☐	☒	A statement on whether measurements were taken from distinct samples or whether the same sample was measured repeatedly
☐	☒	The statistical test(s) used AND whether they are one- or two-sided<br>*Only common tests should be described solely by name; describe more complex techniques in the Methods section.*
☒	☐	A description of all covariates tested
☐	☒	A description of any assumptions or corrections, such as tests of normality and adjustment for multiple comparisons
☐	☒	A full description of the statistical parameters including central tendency (e.g. means) or other basic estimates (e.g. regression coefficient) AND variation (e.g. standard deviation) or associated estimates of uncertainty (e.g. confidence intervals)
☐	☒	For null hypothesis testing, the test statistic (e.g. *F*, *t*, *r*) with confidence intervals, effect sizes, degrees of freedom and *P* value noted<br>*Give P values as exact values whenever suitable.*
☒	☐	For Bayesian analysis, information on the choice of priors and Markov chain Monte Carlo settings
☒	☐	For hierarchical and complex designs, identification of the appropriate level for tests and full reporting of outcomes
☒	☐	Estimates of effect sizes (e.g. Cohen's *d*, Pearson's *r*), indicating how they were calculated

Our web collection on statistics for biologists contains articles on many of the points above.

Software and code

Policy information about availability of computer code

Data collection	Data were required using PCLAMP 10 recording software. Visual stimuli were generated using Matlab2012 and the PsychToolbox.
Data analysis	For patch clamp recordings, data were analyzed using PCLAMP10.4 and custom codes in MATLAB2021b (https://github.com/ww-uchicago/NC-manuscript-2023). For calcium and voltage imaging, Fiji/ImageJ 1.53c and custom codes in MATLAB2021b are used.

For manuscripts utilizing custom algorithms or software that are central to the research but not yet described in published literature, software must be made available to editors and reviewers. We strongly encourage code deposition in a community repository (e.g. GitHub). See the Nature Portfolio guidelines for submitting code & software for further information.

Data

Policy information about availability of data

All manuscripts must include a data availability statement. This statement should provide the following information, where applicable:

- Accession codes, unique identifiers, or web links for publicly available datasets
- A description of any restrictions on data availability
- For clinical datasets or third party data, please ensure that the statement adheres to our policy

Source data are provided with this paper.

April 2023

Research involving human participants, their data, or biological material

Policy information about studies with human participants or human data. See also policy information about sex, gender (identity/presentation), and sexual orientation and race, ethnicity and racism.

Reporting on sex and gender	Not applicable
Reporting on race, ethnicity, or other socially relevant groupings	Not applicable
Population characteristics	N/A
Recruitment	N/A
Ethics oversight	N/A

Note that full information on the approval of the study protocol must also be provided in the manuscript.

Field-specific reporting

Please select the one below that is the best fit for your research. If you are not sure, read the appropriate sections before making your selection.

☒ Life sciences ☐ Behavioural & social sciences ☐ Ecological, evolutionary & environmental sciences

For a reference copy of the document with all sections, see nature.com/documents/nr-reporting-summary-flat.pdf

Life sciences study design

All studies must disclose on these points even when the disclosure is negative.

Sample size	Final sample size was calculated based on preliminary data to have a power of test stronger than 0.8.
Data exclusions	No data are excluded.
Replication	Key experiments were replicated in multiple animals by multiple co-authors over the time course of two years. At least three independent replications with different animals were performed by all experiments. We also replicated published results from previously publications Koren et al., Neuron 2007; Ozaita et al., J. Neurosci 2004.
Randomization	Mice of the same genotype were randomly picked for the experiments. Different visual stimulus conditions were presented in a pseudorandomized sequence generated by Matlab.
Blinding	The investigators were blinded during recording since different stimulus conditions were pseudorandomly generated by Matlab.

Reporting for specific materials, systems and methods

We require information from authors about some types of materials, experimental systems and methods used in many studies. Here, indicate whether each material, system or method listed is relevant to your study. If you are not sure if a list item applies to your research, read the appropriate section before selecting a response.

Materials & experimental systems

n/a	Involved in the study	
☐	☒	Antibodies
☒	☐	Eukaryotic cell lines
☒	☐	Palaeontology and archaeology
☐	☒	Animals and other organisms
☒	☐	Clinical data
☒	☐	Dual use research of concern
☒	☐	Plants

Methods

n/a	Involved in the study	
☒	☐	ChIP-seq
☒	☐	Flow cytometry
☒	☐	MRI-based neuroimaging

Antibodies

Antibodies used	Abcam #ab238432: Rabbit monoclonal anti-mGluR2 antibody (1:500 dilution) and Invitrogen #MA5-27684 Mouse monoclonal anti-

Antibodies used	Kv3.1 antibody (1:500 dilution). Alexa-conjugate secondary antibodies Alexa-488 conjugated goat anti-mouse (Invitrogen Cat # A32723) and Alexa-594 conjugated donkey anti-rabbit (Invitrogen Cat # A-21207) at 1:750 or 1:1000 dilution.
Validation	Validation by Abcam and Invitrogen's supplier information, Ozaita et al., J. Neurosci., 2004, and our own test in mGluR2 knockout mice.

Animals and other research organisms

Policy information about studies involving animals; ARRIVE guidelines recommended for reporting animal research, and Sex and Gender in Research

Laboratory animals	Chat-IRES-Cre mice (129S6-Chattm2(cre)Lowl/J), Chat-CreERT (Chattm1(cre/ERT)Nat) and floxed tdTomato mice (129S6-Gt(ROSA)26Sortm9(CAG-tdTomato)Hze/J) were acquired from the Jackson Laboratory. mGluR2–GFP mouse line was a generous gift from Dr. Marla Feller at University of California, Berkeley. Drd4–GFP mice, in which posterior-preferring DSGCs are selectively labelled, were originally developed by MMRRC in the Swiss Webster background, and were subsequently backcrossed to C57BL/6 background. All strains were backcrossed to the C57BL/6 background in our laboratory and crossed to each other to create the lines used in this study. Mice of ages P21-P40 of both sexes were used.
Wild animals	No wild animals were used in this study.
Reporting on sex	Both sexes were used for data collection.
Field-collected samples	No field-collected samples were used in this study.
Ethics oversight	All procedures to maintain and use mice were in accordance with the University of Chicago Institutional Animal Care and Use Committee (Protocol number ACUP 72247) and in conformance with the NIH Guide for the Care and Use of Laboratory Animals and the Public Health Service Policy.

Note that full information on the approval of the study protocol must also be provided in the manuscript.

Plants

Seed stocks	*Report on the source of all seed stocks or other plant material used. If applicable, state the seed stock centre and catalogue number. If plant specimens were collected from the field, describe the collection location, date and sampling procedures.*
Novel plant genotypes	*Describe the methods by which all novel plant genotypes were produced. This includes those generated by transgenic approaches, gene editing, chemical/radiation-based mutagenesis and hybridization. For transgenic lines, describe the transformation method, the number of independent lines analyzed and the generation upon which experiments were performed. For gene-edited lines, describe the editor used, the endogenous sequence targeted for editing, the targeting guide RNA sequence (if applicable) and how the editor was applied.*
Authentication	*Describe any authentication procedures for each seed stock used or novel genotype generated. Describe any experiments used to assess the effect of a mutation and, where applicable, how potential secondary effects (e.g. second site T-DNA insertions, mosiacism, off-target gene editing) were examined.*
